# Molecular Determinants and the Regulation of Human Cytomegalovirus Latency and Reactivation

**DOI:** 10.3390/v10080444

**Published:** 2018-08-20

**Authors:** Donna Collins-McMillen, Jason Buehler, Megan Peppenelli, Felicia Goodrum

**Affiliations:** 1 BIO5 Institute, University of Arizona, Tucson, AZ 85721, USA; dcoll2@email.arizona.edu (D.C.-M.); jcbuehler@email.arizona.edu (J.B.); mpeppenelli@email.arizona.edu (M.P.); 2 Department of Immunobiology, University of Arizona, Tucson, AZ 85721, USA

**Keywords:** human cytomegalovirus, latency, reactivation, signaling, transcription factors, epigenetic regulation

## Abstract

Human cytomegalovirus (HCMV) is a beta herpesvirus that establishes a life-long persistence in the host, like all herpesviruses, by way of a latent infection. During latency, viral genomes are maintained in a quieted state. Virus replication can be reactivated from latency in response to changes in cellular signaling caused by stress or differentiation. The past decade has brought great insights into the molecular basis of HCMV latency. Here, we review the complex persistence of HCMV with consideration of latent reservoirs, viral determinants and their host interactions, and host signaling and the control of cellular and viral gene expression that contributes to the establishment of and reactivation from latency.

## 1. Introduction

Human cytomegalovirus (HCMV) remains a serious global health burden and has been assigned the highest priority for vaccine development by the National Vaccine Advisory Committee [1]. Infection with HCMV is very common, with seroprevalence among the world’s population ranging from 40–99% depending on geographical location and socioeconomic status [2]. HCMV infection of healthy individuals routinely goes undiagnosed, as symptoms are ambiguous and relatively mild; however, in the absence of adequate host adaptive immunity, infection poses a life-threatening disease risk [3]. HCMV is an important opportunistic pathogen among acquired immune deficiency syndrome (AIDS) patients [4] and those undergoing immunosuppressive therapies for the treatment of cancer [5]. HCMV is also the leading cause of infectious complications following solid organ [6] or stem cell transplantation [7,8], manifesting as interstitial pneumonia, gastroenteritis, retinitis, hepatitis, graft failure, and death [9]. Additionally, HCMV is the most common infectious cause of birth defects and is also more prevalent than some well-known non-infectious congenital conditions, such as Down syndrome or fetal alcohol syndrome [10]. Congenital HCMV infection of the immune naïve fetus may result in mild to severe hearing loss, cognitive impairment, microcephaly, and/or cerebral palsy [10].

Much of the pathogenesis associated with HCMV infection can be attributed to the virus’s ability to establish a persistent life-long infection in the host through latency. In its latent state, the viral genome is maintained in the host cell without active replication or the production of new viral progeny but with the capacity to reactivate viral replication from latency in response to changes in the host cell. Thus, HCMV has evolved a sophisticated persistence strategy whereby the virus reaches an armistice with the host by limiting viral replication and pathogenesis to avoid immune clearance. This strategy includes periodic reactivation events that, despite being well-controlled by the immune system, still allow for the production of viral progeny and their spread to additional hosts. Serious disease arises when an uncontrolled reactivation event occurs as a result of immunosuppression in an otherwise healthy host or, in the case of congenital infection, leads to transmission of the virus to an immune naïve host [11]. Indeed, a recent analysis of congenital HCMV infections in the United States from 1988 to 1994 revealed that only one in four resulted from primary infection of the mother during pregnancy [12], highlighting the importance of reactivation from latency (or re-infection) in seropositive pregnant women. In addition, mounting evidence suggests that the health cost of asymptomatic persistence in otherwise healthy individuals increases as we age, with HCMV emerging as a risk factor for the development of age-related pathologies, such as cardiovascular disease [13,14,15,16], immune dysfunction [17,18], and frailty [19,20,21].

HCMV has a broad cellular tropism and infects a number of cell types during primary infection; however, the outcome of infection varies widely and is largely cell type-dependent. Some cell types, such as fibroblasts and smooth muscle cells, support robust viral replication and provide a platform for high levels of viral proliferation from which the virus is likely eventually cleared [22]. Epithelial and endothelial cells support a chronic or “smoldering” infection that presumably elicits a subtler immune response, resulting in low-level virus shedding that facilitates both interhost (e.g., oral epithelial cells) and intrahost (e.g., endothelial lining of the blood vessels) transmission of the virus [22,23]. Undifferentiated hematopoietic cells, such as CD34+ human progenitor cells (HPCs) and CD14+ monocytes, do not support a replicative infection and serve as a reservoir for HCMV latency as well as a platform for viral dissemination throughout the host [24,25,26,27,28,29,30,31]. Latent HCMV can then reactivate gene expression when the infected cell differentiates along the myeloid lineage toward a macrophage or a dendritic cell [32,33,34,35,36].

## 2. HCMV Latency Reservoirs

**Figure 1:**
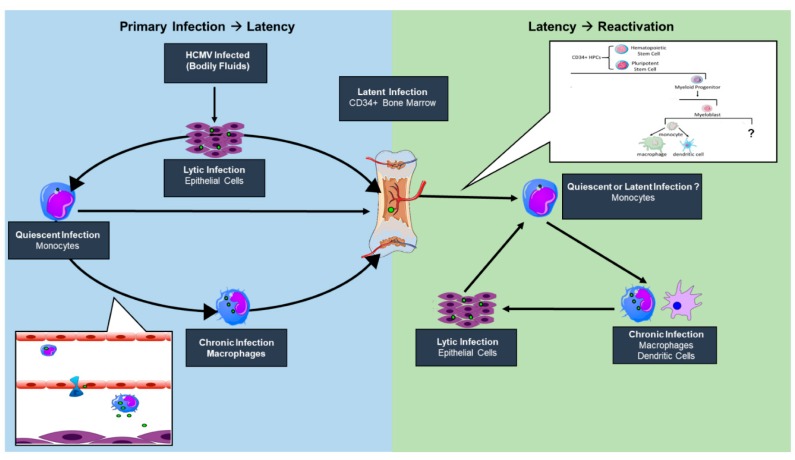
A model of human cytomegalovirus (HCMV) infection reservoirs. An initial round of lytic replication occurs in mucosal epithelial cells following virus exposure. Circulating monocytes are infected through contact with an infected epithelial cell and induce monocyte-to-macrophage differentiation and extravasation to peripheral tissues, including the bone marrow where a latent infection is established. Upon a reactivation signal, HCMV can induce the differentiation and mobilization of latently infected CD34+ bone marrow cells. While not entirely defined, HCMV may stimulate CD34+ human progenitor cell (HPC) differentiation down a monocytic lineage with an eventual terminal differentiation state of an infected macrophage or dendritic cell. These terminally differentiated cells allow for the reseeding of epithelial cell layers within peripheral organ sites.

HCMV is highly species-specific but its cell tropism within the human host is vast, infecting endothelial, epithelial, fibroblasts, neuronal, monocytes/macrophages, granulocytes, and smooth muscle cells [37]. This broad cell tropism allows for the establishment of persistent infection, with dissemination beginning when a seronegative individual comes into contact with bodily fluids, such as tears, saliva, urine, semen, or breast milk, of an infected individual [38,39,40]. An initial round of lytic replication occurs in mucosal epithelial cells, where circulating peripheral blood CD14+ monocytes (PBM) in contact with infected epithelial cells may become infected (Figure 1). HCMV induces monocyte-to-inflammatory macrophage differentiation and survival of newly infected PBMs by activating EGFR/PI3K/Akt and integrin/src cellular signaling pathways during entry in the absence of de novo viral gene expression [41,42,43,44,45,46,47]. HCMV primes differentiating PBMs via the activation of the PI3K/NF-kappaB pathway to extravasate from the circulation to peripheral organ sites [48], establishing a “chronic infection” or a low-grade lytic infection reservoir in tissue-resident macrophages and neighboring epithelial layers. Further, monocytes can travel directly to the bone marrow and infect CD34+ hematopoietic stem cells, a major latency reservoir, establishing a life-long latent infection [49,50]. A direct infection of circulating CD34+ cells by mucosal epithelial cells then a subsequent migration to and seeding of the bone marrow is possible but remains elusive. HCMV can remain latent for extended periods of time within the hematopoietic cells; however, reactivation can occur following immune insult, allogenic stimulation, or differentiation signals [34] (Figure 1).

The state of knowledge in the field supports the notion that reactivation has no singular signal but is a multifaceted event where the virus is an active participant. We propose that a nexus of viral microRNAs and proteins, including ULb’ or tegument proteins, and cellular signaling play a role in the switch from latent to lytic infection in the bone marrow myeloid niche [51,52,53,54]. The relative contributions of viral and host factors in initiating or sustaining reactivation are far from clear. However, it appears that changes in the immune, homeostatic, or stress state at the level of the host or infected cell can result in changes in signaling that stimulate changes in viral gene expression, forging the path towards successful replication. In addition to altering viral activity, changes in host signaling associated with either latency or reactivation from latency have profound implications for hematopoietic differentiation. HCMV is known to be myelosuppressive following stem cell transplantation. Preferential differentiation of primary CD34+ hematopoietic progenitors into immunosuppressive monocytes following reactivation in a STAT-3-dependent manner has been demonstrated in vitro [55]. Whether these immunosuppressive monocytes are a “secondary latency” state or a “quiescent infection” still remains to be determined, as viral gene expression and the requirement of an external stimulus to promote differentiation has yet to be determined. Allogenic stimulation of monocytes [34] or monocyte-to-macrophage differentiation [35,36] stimulates HCMV reactivation, strongly suggesting that myeloid lineage differentiation and activation pathways are connected with HCMV reactivation. Further, differentiation of CD34+ progenitor cells to mature dendritic cells results in reactivated viral lytic gene expression and infectious virus production [56]. Defining alterations in differentiation between cells infected as progenitor cells or circulating monocytes would contribute to our understanding of viral persistence and the potential for reseeding peripheral tissue sites following bone marrow mobilization (Figure 1). However, there has been no study to date that comprehensively addresses the possibility of other cellular latency reservoirs within the host. While these may exist, our review will focus on work done to understand establishment and maintenance of or reactivation from latency in hematopoietic cell model systems.

## 3. HCMV Latency Models

In vitro systems have been vital for the study of HCMV latency for two reasons. First, the number of cells harboring latent virus in the infected host is extremely low (between 1:10,000 to 1:100,000 cells carry viral genomes) [57], and there is currently no way to identify and enrich for these cells. Second, CMVs are highly restricted in their tropism, so the human virus is challenging to study outside of cultured cells. While a number of cell culture models exist, these models present their own challenges associated with the difficulty in culturing primary hematopoietic cells and the limited utility of immortalized or transformed cells for the study of HCMV.

### 3.1. Primary Progenitor Cells

CD34+ HPCs are a heterogeneous subpopulation of cells ranging from pluripotent stem cells to cells in the earliest stages of commitment to myeloid or lymphoid lineage. Ex vivo culture should be carefully considered given that our understanding of the complexity of molecular events that induce or block differentiation is still very limited. This limitation creates a risk that ex vivo culture may result in artefact given that CD34+ HPCs are so prone to differentiate and may do so aberrantly given their culture conditions. To address this, stromal cell co-culture and stromal-free systems have been developed in an attempt to maintain the physiological relevance of cultured CD34+ HPCs. In the case of stromal cell support cultures, stromal cell clones recapitulate the hematopoietic microenvironment in vitro. These stromal cell cultures release a physiologically balanced milieu of cytokines and have been demonstrated to maintain hematopoietic progenitor cell phenotype and support stem cell self-renewal such that they retain their ability to repopulate the hematopoietic system in mice following xenotransplantation [58]. We have demonstrated the ability of these cultures to maintain latently infected cells [24,59,60,61]. Cell-free culture systems for CD34+ HPCs are also widely used that require the addition of cytokine cocktails to maintain cell phenotype and viability or promote differentiation down a specific lineage [62,63,64,65]. In all in vitro systems, reactivation is stimulated by inducing myeloid differentiation, often with fibroblast co-culture to detect virus produced from reactivation [24,56].

### 3.2. Primary Monocytes

Monocytes are a clear reservoir of latent virus and are critical to viral dissemination in the host. However, it remains unclear if direct infection of monocytes in vitro results in a bona fide latent infection [26,64] or a “quiescent infection” during which viral genomes are carried, but expression of viral genes is delayed and is associated with cytokine stimulation (e.g., granulocyte macrophage-colony stimulating factor, GM-CSF) and macrophage differentiation [32,34]. The Yurochko group demonstrated that monocyte-to-macrophage differentiation occurs in vitro at approximately 3 weeks post infection without the addition of GMCSF or MCSF as survival signals [66]. During this 3-week period, no lytic gene expression is detected [67]. It remains to be determined whether HCMV induces a de novo cellular signal to drive monocyte-to-macrophage differentiation in the absence of an external stimulus. If so, then monocytes that become infected in the circulation may exist as a distinct latency reservoir from monocytes that were infected as CD34+ HPCs and simply retained viral genomes through their differentiation. This primary “quiescent infection” model in monocytes has been used to study early entry and signaling events required for dissemination during HCMV infection, which is not possible in cells lines [41,42,67,68,69,70,71].

### 3.3. Cell Lines

Cell lines have long been sought as a model for HCMV latency as they offer the advantages of homogeneity, ease of cost and culture, and enhanced availability relative to primary cell systems. However, many cell lines do not faithfully recapitulate all the hallmarks of latency (e.g., the ability to produce progeny virus upon differentiation into a permissive cell type), leaving primary cell cultures as the gold standard for studying HCMV latency. Human monocytic leukemia cells (THP-1) have long been used to model the latent infection. In these cells, transcriptional reactivation from latency is achieved following treatment with phorbol ester [72], as subsequent viral immediate early (IE) genes [73] and early gene products (DNA polymerase) are expressed [74,75]. THP-1 cells have been successfully utilized for the study of viral gene expression [76,77], viral manipulation of cellular transcription factors [78,79], histone modification [80,81], and chromatin remodeling [73]. The CD34+ Kasumi 3 leukemic cell line and embryonic cell lines (ESC) are alternative in vitro models that harbor latent genomes and reactivate producing viral progeny [82,83]. Kasumi 3 have been used to study FAS ligand-mediated apoptosis through cellular IL-10 [84]. ESCs are typically differentiated into a neural precursor differentiation to study HCMV infection during neural development [85].

The NTERA-2 (NT2) cell line is a pluripotent human embryonal carcinoma cell line that can be differentiated into neurons following treatment with retinoic acid (RA) [86]. These cells have been used as a model for HCMV latency and reactivation, as HCMV establishes a latent infection in NT2 neuronal precursors that is reactivated following RA-stimulated differentiation [87]. While these cells do not model latency in myeloid lineage cells, they may serve as a model for latency in neuronal progenitor cells in vivo [88,89,90].

### 3.4. Animal Models

Human CMV is restricted in tropism to primary human cells. This barrier has complicated understanding CMV latency and the immune responses to infection. Murine CMV has been an invaluable tool for understanding latency, but it differs from the human virus in a number of significant ways. Namely, the smaller murine CMV lacks the ULb’ region of the genome that has been shown to encode a number of viral proteins important to latency and reactivation [51,91,92,93,94,95,96]. A humanized mouse model (huNSG) has been developed that supports a latent infection with the human virus in human hematopoietic cells, and recapitulates many of the phenotypes demonstrated in vitro [97]. UL136, a ULb’ gene, expresses 5 isoforms that have both replication-repressive (23- and 19-kDa isoforms) and -promoting (33- and 26-kDa) functions and contribute to latency and reactivation in CD34+ HPCs, respectively [94,95]. The in vitro phenotypes of the UL136-mutant viruses lacking specific isoforms are largely recapitulated in huNSG mice [94]. The 25-kDa UL136 isoform differs in its in vitro and in vivo phenotypes; mutation of this isoform results in a virus more prone to reactivate in vitro but fails to reactivate in vivo. This finding suggests that these systems may allow differentiation of phenotypes important for infection in the host. Further, binding of HCMV UL7 to Fms-like tyrosine kinase 3 receptor (Flt-3R) promotes differentiation in the huNSG mouse model, and is required for reactivation in CD34+ HPC and CD14+ monocyte models as well as in huNSG mice [98]. These cornerstone studies establish the importance of the humanized mouse model in defining viral or host factors important to latency and in understanding the impact of HCMV latency on signaling, immune responses, and hematopoiesis.

## 4. CMV-Mediated Control of Host Signaling for Latency/Reactivation

CMV is a master manipulator of the cellular environment with the seemingly simple goal of persisting within its host. While the virus infects a broad range of cells, one commonality across cell types is that CMV manipulates a variety of signaling pathways to reconfigure the homeostatic environment for viral objectives. However, our understanding of CMV-mediated manipulation of host signaling has been largely generated through studies in fibroblasts during active viral replication, while a significantly smaller portion of work has been targeted toward cell types that support latency. For the purposes of this review, our focus will be on the body of work pertaining only to latency models to highlight the importance of signaling during latency and the necessity for future work in this area.

### 4.1. CMV Initiation of EGFR and Integrin Signaling: Starting off on the Right Receptor

**Figure 2:**
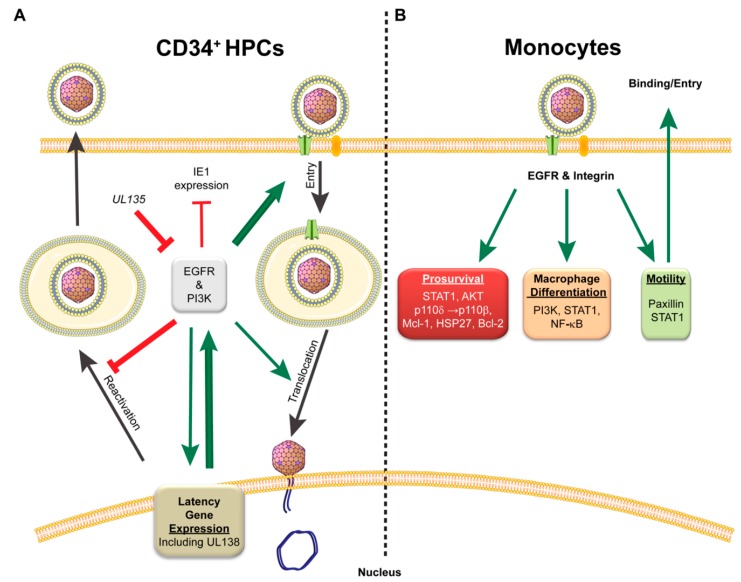
Importance of epidermal growth factor receptor (EGFR) and integrin signaling during CMV latent infection. (**A**) EGFR signaling is important for CMV entry, translocation to the nucleus, and to establish latency. Attenuation of EGFR signaling by the UL135 proteins or inhibitors disrupts this pattern and contributes to reactivation of CMV replication. Thickness of arrows and lines depicts strength of supporting studies; (**B**) In monocytes, CMV binding and entry via EGFR and integrin receptors results in changes in signaling to promote cellular survival, macrophage differentiation, and motility to promote virus dissemination in the infected host. Red T bars indicate inhibition. Green arrows indicate activation.

Cytomegalovirus infection is a well-choreographed series of events to take over a cell from initial binding and entry to successful virus progeny production or the establishment of latency, all of which contributes to life-long persistence within the host. To achieve early control of cells destined for latency, CMV binds signaling receptors, such as epidermal growth factor receptor (EGFR), platelet-derived growth factor receptor (PDGFR), and integrins, on the surface of the cell to facilitate viral entry and to initiate cellular signaling [42,52,99,100]. While CMV does enter through PDGFR in fibroblasts, endothelial cells, and epithelial cells, little is known about its role in signaling during infection in latency cell types, so focus will be given to EGFR and integrins for this section. The use of both EGFR and integrins as entry receptors initiates downstream signaling events that can prime the cells for the establishment of a latent infection prior to the virus even entering the cell (Figure 2). The activation of EGFR allows the virus to manipulate cellular proliferation, differentiation, migration, survival, innate immunity, and DNA repair [101,102,103,104,105], while integrin signaling regulates many of the above functions but also interacts with and influences intra- and intercellular homeostatic signaling by tyrosine kinase receptors, such as EGFR (Reviewed in [106,107,108]).

In CD34+ hematopoietic progenitor cells (HPCs), EGFR and integrin signaling is integral for CMV entry and the establishment of latency [52,68,69,70]. Kim et al. demonstrated that blocking EGFR signaling, either through chemical inhibition or with an interfering antibody, retains virions at the cell surface with EGFR and diminishes entry into CD34+ HPCs [52]. Inhibition of phosphatidylinositol-4,5-bisphosphate 3-kinase (PI3K), downstream of EGFR, also inhibits entry of CMV into CD34+ HPCs. EGFR trafficking is required for translocation of the viral genome to the nucleus to initiate CMV infection, and inhibition of EGFR signaling shortly after entry significantly delays gene expression. Interestingly, CMV infection of CD34+ HPCs results in a transient increase in surface expression of EGFR early on in infection [51]. This is in sharp contrast to infection in fibroblasts, where EGFR levels are reduced 50–70% compared to uninfected controls, and inhibition of EGFR kinase activity further enhances virus replication [51]. This cell-type-dependent regulation of EGFR supports a possible requirement for EGFR signaling during CMV infection of CD34+ HPCs for the establishment of latency, and loss of EGFR or PI3K signaling enhances reactivation potential during infection [51]. Similar results were seen in HSV-1 and pseudorabies virus infection when PI3K signaling was inhibited [109,110]. Further, inhibition of EGFR by AG1478, a chemical inhibitor of EGFR kinase activity, increases IE1 gene expression while decreasing the UL138 latency determinant in CD34+ HPCs [52]. The changes in EGFR signaling in CD34+ HPCs may explain some of the infection-induced alterations in myeloid differentiation, as levels of the inflammatory cytokine interleukin-12 are increased during CMV infection and further exacerbated by inhibition of EGFR signaling [52]. Collectively, this work demonstrates a pivotal role for EGFR and virus-mediated control of EGFR signaling in regulating replicative and latent states during HCMV infection.

The intertwining of integrin, specifically β1 and β3, and EGFR signaling has been shown to be critical for HCMV infection of monocytes [69,70]. Virus entry into monocytes also requires the pUL128–131 pentameric complex proteins, which activate the integrin-c-Src signaling pathway to alter the actin cytoskeleton [70]. Within 5 min following entry, infection further alters EGFR-dependent Stat1 signaling to generate pro-survival and motility signals [99]. CMV maintenance of pro-survival signaling and promotion of cellular motility is driven through AKT and STAT1 pathways and represents a major viral target during monocyte infection [41,42,67,68,99]. Within 15 min of gB binding to EGFR on a monocyte, CMV increases AKT signaling in a PI3K-dependent manner [41,67]. The activation of Akt occurs through shifting PI3-kinase preference from p110δ to p110β and by stimulating SH2-domain-containing inositol 5-phosphatase 1 (SHIP1)-mediated conversion of PI3P to PI2P. These alterations to signaling result in the maintenance of AKT activation for up 72 h, allowing for the monocytes to survive beyond the typical 48-h survival gate [41]. This non-canonical activation of AKT signaling during HCMV infection preferentially activates mTOR to promote the expression of myeloid cell leukemia sequence 1 (Mcl-1) and heat shock protein 27 (HSP27), which prevents the cleavage of caspases and the initiation of apoptosis [42]. As CMV infection progresses, Bcl-2 is required to maintain the anti-apoptotic state to counter an upregulation in Bax expression [111]. These changes in signaling restructure the cytokine environment to increase motility and migration [69]. Enhanced survival and motility of infected monocytes aids dissemination of CMV throughout the infected host to tissues [112]. Additionally, CMV-mediated activation of PI3K and STAT1, as well as NF-κB, also promotes monocyte-to-macrophage differentiation, which is necessary to promote viral replication and dissemination within and between hosts [44,99]. Together, the body of work demonstrates that CMV manipulates EGFR, integrins, and their downstream pathways for infection, persistence, and dissemination in the host.

### 4.2. CMV Manipulation of the Cellular Environment for Establishment and Maintenance of Latent Population

**Figure 3:**
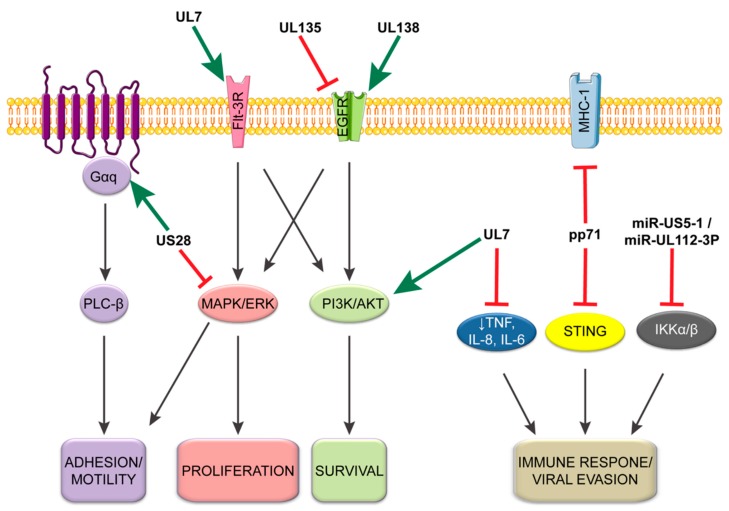
Viral regulation of cellular signaling during latency. CMV utilizes a number of viral proteins and miRNAs during latent infection to alter the signaling environment within the cell to regulate the latent infection. This model demonstrates how each viral component either inhibits (red cross) or promotes (green arrow) cellular signaling events during a latent infection. As depicted, a number of viral factors target similar pathways demonstrating their necessity in the maintenance of and reactivation from latency.

Herpesvirus latency is associated with profound changes in cell signaling to modulate survival, sensing, and differentiation [53,113,114]. In the case of HCMV infection in monocytes, these changes include increasing MCP-1 to enhance cellular migration, promotion of immune response genes, increase protein and lipid metabolism, and a dramatic shift in the differentiation profile toward macrophages [115,116,117]. Some of these changes are the result of signaling outcomes initiated during entry, as described above, but many other mechanisms exist to stabilize and maintain a latently infected population. This subsection will focus on those other pathways (Figure 3).

Like all viruses, CMV must evade the cellular intrinsic detection of foreign DNA. To accommodate this, pp71, a tegument protein encoded by CMV UL82, binds to the stimulator of interferon genes (STING) to inhibit its antiviral response [118]. The interaction between the pp71 tegument protein and STING prevents the translocation of STING from the ER to microsomes, subsequently preventing the recruitment and phosphorylation of IRF3 and TBK1. Loss of pp71 results in increased IFNβ1, ISG56, TNF, and IL-6 in THP-1, macrophage, and dendritic cells as well as an increase in ISG15 and IL-1β in THP-1 cells. This pp71-mediated evasion is also supported by an alternate function of the proteins, which decreases cell surface expression of major histocompatibility complex 1 (MHC-I) during CMV infection to prevent detection by the adaptive immune system [119]. Together, these results suggest that pp71 plays an important role in modulating CMV immune evasion following entry across cell types and might be important for establishing latency.

The UL133–UL138 locus encoded within the ULb’ region of the HCMV genome is dispensable for replication in fibroblasts but required for latency in hematopoietic cells. The ULb’ region is present in clinical and low-passage strains of the virus (e.g., Merlin, TB40/E, FIX), but lost during serial passage of all laboratory strains (e.g., AD169, Towne short) [120,121,122]. Four genes are encoded from this locus: UL133, UL135, UL136, and UL138 [91,123]. Replication-suppressing functions important for the establishment or maintenance of latency have been attributed to UL133 and UL138 [91,92,93]. By contrast, UL135 is important for reactivation from latency and functions, in part, by overcoming the growth-suppressive function of UL138 [124]. UL136 is more complicated in that it is expressed as five protein isoforms differing only in their amino terminal ends [95]. UL136 isoforms are expressed with later kinetics relative to the other proteins encoded by the UL133–UL138 locus, at least in fibroblasts. The two large membrane-bound UL136 isoforms are required for reactivation in CD34+ HPCs and in humanized mice, whereas the two small soluble isoforms suppress replication for latency [94]. Given the later expression kinetics of UL136 and the seemingly overlapping function of the isoforms with UL136 and UL135 in latency, a model can be envisioned whereby UL138 and UL135 contribute to the establishment of replicative or latent states, respectively. UL136 follows, perhaps in response to reactivation cues, and interplay between the UL136 isoforms facilitates maintenance of latency or reactivation. This model, while intriguing, has yet to be validated and much remains to be understood about the mechanisms by which UL136 isoforms mediate latent versus replicative states.

While much remains to be understood about the mechanisms by which UL133–UL138 proteins work to regulate states of latency and replication, the opposing functions of UL138 and UL135 in mediating latency and reactivation, respectively, have been attributed to their opposing regulation of epidermal growth factor signaling (EGFR) [51]. EGFR or downstream PI3K signaling pathways inhibit replication in fibroblasts following viral entry, and HCMV infection results in the downregulation of EGFR early in infection [51,125]. Consistent with this relationship, inhibition of EGFR and PI3K facilitates reactivation when combined with a differentiation stimulus. UL138 has been shown to upregulate EGFR at the cell surface, whereas UL135 stimulates the turnover of EGFR from the cell surface [51]. UL135 stimulates EGFR turnover by interacting with the host adapter proteins CIN85 and Abi-1, which are important for the intracellular trafficking of EGFR toward the lysosome [96]. It will be important to determine how CMV modulation of EGFR trafficking affects downstream signaling to affect latency and replication and how the function of UL133 and the UL136 isoforms contribute to this regulation. Receptor tyrosine kinase signaling, and specifically EGFR or analogous pathways, has been shown to be important for latency in other herpesvirus infections, including HSV-1, EBV, and KSHV [126,127,128].

The viral G-protein-coupled receptor (vGPCR) US28 also plays an important role in modulating the host environment in latency. During lytic infection in fibroblasts, US28 promotes MAPK, NF-κB, and CREB signaling to drive MIEP activity, which is facilitated by US28 binding a variety of chemokines and G proteins [129,130,131,132,133]. In the context of latency in primary CD34+ HPCs or the Kasumi-3 cell line, US28 is highly expressed relative to the immediate early IE1 gene and is required for the establishment of latency [134]. However, in the context of latency and in contrast to lytic infection, US28 is reported to attenuate NF-κB and MAPK signaling [134,135]. In the absence of US28, infected CD34+ HPCs have increased IE1 expression prior to and following a reactivation stimulus [134]. Additionally, US28 promotes cellular adhesion of monocytes on endothelial cells by activating Gαq/PLC-β signaling, which is dependent on G-protein coupling and independent of chemokine binding [136]. These findings demonstrate a dichotomy in US28 function and cell-type-specific infection outcome. It will be important to determine how the function of US28 switches to support latent versus replicative states in the context of reactivation from latency.

Another important aspect of persistence is the ability of viruses to assuage inflammatory and immune responses. CMV induces expression of the anti-inflammatory cytokine interleukin-10 (IL-10) to block FAS-mediated apoptosis of latently infected CD34+ HPCs [84]. By increasing IL-10, CMV promotes the expression of the potent FAS/TNFR1 PEA-15 [84,137]. Knockdown of either IL-10 or PEA-15 increased apoptotic susceptibility of latently infected CD34+ HPCs [84]. The anti-inflammatory state is further reinforced by the CMV miRNAs miR-US5-1 and miR-UL112-3P targeting IKKα and IKKβ to decrease IL-1β and TNF-α responsiveness [138]. These viral miRNAs target the 3’ UTR of IKKα and IKKβ in response to NF-κB signaling. Loss of miR-US5-1 and miR-UL112-3P results in increased NF-κB signaling and increased IL-6, CCL5, and TNF-α in THP-1 cells. Such increase in IL-6 particularly has been demonstrated to increase CMV reactivation from latently infected dendritic cells through ERK-MAPK pathways by promoting IE gene expression [139].

In addition to UL135, CMV UL7 also functions to regulate signaling and is required for reactivation. UL7 is secreted and functions as a ligand, sharing many similarities to the signaling lymphocyte-activation molecule (SLAM) CD229 [140]. Like many SLAM family members, UL7 is able promote leukocyte adhesion, which it does independent of SLAM family member interaction. UL7 activates PI3K/AKT and MAPK/ERK pathways by serving as a ligand for Fms-like tyrosine kinase 3 receptor (Flt-3R) in CD34+ HPCs [98]. UL7 suppresses the proinflammatory response by decreasing TNF, IL-8, and IL-6 expression in myeloid cells and promotes myelopoiesis and differentiation of monocytes and CD34+ HPCs. Further, in HCMV infection UL7 is required for HCMV reactivation from latency in both in vitro CD34+ cells and in vivo humanized mice as well as G-CSF-induced myelopoiesis in humanized mice. These results suggest that UL7 is a potent viral autocrine regulator of reactivation and hematopoietic differentiation. It will be interesting to understand how the UL7 promotion of PI3K/AKT and MAPK/ERK signaling contributes to reactivation in light of other studies which show that EGFR and PI3K signaling contribute to latency. These studies may indicate the importance of timing in the regulation of host signaling.

The perversion of cellular signaling pathways during CMV infection is critical to ensure cell survival and establish an intracellular environment conducive to latency and to avoid immune detection. There is still a substantial body of work that needs to be performed to fully define the degree to which CMV controls latently infected cells. This includes understanding the subtle signaling differences in the various latency models, such as monocytes versus dendritic cells versus CD34+ HPCs, and how these may change over time during latent infection. Additionally, there are many signaling pathways that lack sufficient data in latency cell types to determine how CMV interferes with their normal function.

In going forward, WNT is an important signaling pathway to consider in CMV latency as it is critical to the control of latency in other herpesviruses. Herpesviruses typically inhibit WNT signaling during replication and promote WNT signaling for the establishment of latency (Reviewed in [141]). Both EBV and KHSV target Glycogen synthase kinase 3 α/β (GSK-3) of the WNT destruction complex to prevent the degradation of β-catenin during latency [142,143,144]. The EBV LMP2A promotes AKT signaling to inhibit GSK-3 destruction of β-catenin [142], while the KSHV LANA interacts with the GSK-3 and sequesters it in the nucleus [143,144]. Additionally, the EBV LMP-1 increases β-catenin levels by blocking its ubiquitination [145,146,147]. The multifaceted approach evolved by γ-herpesviruses to maintain WNT signaling underscores the importance of this pathway to viral latency. Of the α-herpesviruses, the bovine herpesvirus 1 (BHV1) ORF2 protein promotes WNT signaling through interactions with high-mobility group AT-hook protein (HMGA1), mastermind-like protein 1 (MAML1), and AKT3 to promote WNT signaling [148,149,150]. Interestingly, reactivation of BHV1 results in the production of Dickkopf-1 (DKK1) and secreted Frizzled-related protein 2 (SFRP2), which antagonize WNT signaling [148]. This suggests that controlling WNT signaling can serve as a molecular switch between latency and reactivation that may be important across herpesviridae. In CMV infection, lytic replication inhibits WNT signaling by increasing β-catenin turnover or sequestration in the viral assembly compartment [151,152]. However, despite this, inhibition of WNT signaling has been shown to inhibit immediate early gene expression and replication [153]. It will be important to define the role of WNT signaling in modulating states of replication and latency in CMV infection.

## 5. Cellular Control of the Viral Gene Expression Program for Latency/Reactivation

During replicative infection, such as in fibroblasts, the HCMV genome is transcribed in a temporal cascade of three kinetic gene classes designated as immediate early (IE), early (E), and late (L) [154,155]. The major immediate early (MIE) genes are expressed first following activation of the major immediate early promoter (MIEP) by cellular transcription factors and viral tegument components [156]. The MIE gene products (IE1-72 kDa and IE2-86 kDa) transactivate viral early gene promoters [157,158], leading to viral DNA replication and expression of the viral late genes [159]. Because initial activation of the MIEP occurs without a requirement for de novo synthesis of viral proteins, it represents a crucial control point where the cellular environment can exert control over the viral replicative cycle. As such, regulation of the MIEP has been the focus of numerous studies aimed at unravelling the virus–host interactions that govern viral gene expression during latency and reactivation. The MIEP is chromatinized and silenced during latency [56,81,160]. This section reviews our current knowledge of how the MIEP is regulated to control the transition between latent and replicative states of infection.

### 5.1. Cellular Transcription Factors

**Figure 4:**
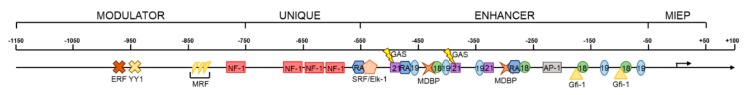
Diagram of cellular transcription factor binding to the HCMV major immediate early promoter (MIEP). The major immediate early core promoter, enhancer, unique region, and modulator are shown. Relative locations of binding sites for activating and repressive cellular transcription factors are shown. Repeat elements are labeled with the number of base pairs repeated in each sequence. The 18-bp repeats bind NF-κB/rel, the 19-bp repeats bind CREB/ATF, and the 21-bp repeats bind SP-1, YY1, and ERF.

The MIEP consists of a core promoter (−65 to +3 nucleotides from the MIE transcription start site), an upstream enhancer (−520 to −65 nucleotides), a unique region (−780 to −610 nucleotides), and a modulator (−1145 to −750 nucleotides) (Figure 4). The core promoter is sufficient for low-level transcription of MIE genes [161]. The enhancer element, which augments transcription from the MIE locus [162,163,164], contains a number of small repeat sequences (18-bp, 19-bp, and 21-bp) that function as binding sites for activating cellular transcription factors [161,162,163,164,165,166]. The 18-bp repeats bind NF-κB/rel [167], the 19-bp repeats bind CREB/ATF [168], and the 21-bp repeats bind Sp-1 [169]. Although the cyclic AMP (cAMP) response element binding (CREB) protein sites and the NF-κB sites have received the most attention, additional binding sites for activators of transcription have been identified in the enhancer, including sites for AP-1 [170], retinoic acid (RA) [171,172,173], serum response factor (SRF)/Elk-1 [174], and gamma-interferon activating sequence (GAS) [175]. Binding sites for repressive transcription factors methylate DNA binding protein (MDBP) [176] and growth factor independence 1 (Gfi-1) [177] have also been identified in the enhancer, and subsequent studies found that the 21-bp repeats also bind the repressive transcription factors Yin Yang-1 (YY1) [178] and Ets-2 repressor factor (ERF) [179], which likely reflects the repressive effect of this region in undifferentiated hematopoietic cells [180]. The unique region contains a cluster of binding sites for the activating transcription factor nuclear factor-1 (NF-1) [181,182]. The modulator contains binding sites for the repressors ERF [179] and YY1 [178], formerly identified as modulator binding factors (MBFs) 1 and 4, respectively [180,183], and modulator recognition factor (MRF) [184]. The enormous number of cellular transcription factors potentially impacting MIEP activity makes for a complex landscape of regulation.

In transient transfection assays, the modulator was shown to alter transcription from the MIE locus in a differentiation-dependent manner. The modulator’s effects are repressive in undifferentiated THP-1 and NT2 cells; however, the modulator stimulates MIE gene expression in permissive fibroblasts [183,184,185,186]. Because undifferentiated progenitor cells harbor a latent HCMV infection, whereas their differentiated counterparts support viral replication, the cellular transcription factors binding the modulator appeared to be ideal candidates for determining whether the virus enters a replicative or latent state of infection. In addition, a number of these studies in non-permissive cell types seemed to indicate that the 21-bp repeats in the enhancer region, in addition to the modulator, are responsible for the inhibition of MIEP activity in undifferentiated cells [180,185,186], consistent with binding of the repressive factors YY1 [178] and ERF [179] to the 21-bp repeats. Given these findings and the discovery that the modulator element showed changes in DNAseI hypersensitivity following differentiation to a cell type that is permissive for viral replication [187], it was hypothesized that differential binding of cellular transcription factors in latency-associated versus permissive cells played a key role in determining IE expression in each cell type. Emphasis was put on examining the balance of activating versus repressive transcription factors present in replication-permissive cells compared to latency-associated cell types, with unclear results.

Differentiation of non-permissive cells into a permissive phenotype correlates with increases in the expression of transcription factors that stimulate transcription from the MIEP by binding the enhancer region. For example, stimulation of the cAMP/PKA signaling pathway [188,189] or addition of phorbol 12-myristate 13-acetate (PMA) [190], which would activate CREB [188,189] or CREB and NF-κB [190], increases IE expression in latently infected NT2 cells, and upregulation of NF-kB during myeloid differentiation also contributes to increased IE expression [191]. However, the stimuli used to activate cell signaling pathways in these assays would also induce additional cellular changes that accompany differentiation and could trigger viral reactivation by mechanisms other than upregulation of activating transcription factors. Transcription factors that repress the MIEP in transfection assays (e.g., modulator binding factor 1 (MBF1) or ERF [179,180,183], MRF [184], YY1 [178], and Gfi-1 [177]) appear to be expressed at higher levels in undifferentiated latency-associated cell types when compared to cell types that are permissive for viral replication; however, differentiation of these cells results in little change in the mRNA expression of either YY1 or ERF [179]. It remains possible that the differences in YY1 and ERF expression levels following differentiation are the result of post-translational regulation and not upregulation of the genes following cell differentiation. Despite similar expression levels of YY1 and ERF both prior to and following differentiation, YY1-mediated [178] and ERF-mediated [179] repression of the MIEP are decreased following differentiation stimulus. Importantly, in contrast to the transient transfection studies, deletion of the modulator in recombinant viruses does not have a measurable effect on IE1 and IE2 transcription during experimental infection of permissive fibroblasts or undifferentiated NT2 or THP-1 cells [192], suggesting that additional viral factors may also play a role in repressing MIE viral gene expression. Going forward, it will be important to understand how cellular transcription factors regulate the viral genome to affect states of latency or reactivation. This will be important to understand not only in the major immediate early region, but also throughout the genome, and particularly with regard to latency-associated genes.

### 5.2. Chromatinization

YY1 [178] and ERF [179] binding to the MIE enhancer and modulator regions and their repression of MIE gene expression has been attributed to histone deacetylase (HDAC)-mediated chromatin remodeling of the MIEP enhancer region to silence IE gene expression [193,194]. An opposite function been shown for CREB, which acts cooperatively with mitogen and stress-activated kinases (MSKs) to initiate chromatin remodeling at the MIEP required for expression of IE genes and viral reactivation [195]. Therefore, a major area of focus in latency and reactivation across the herpesvirus field has become regulation of viral gene expression through epigenetic modifications by the cellular machinery [196].

CMV gene expression is controlled by histone modifications (acetylation or methylation), and the CMV genome itself is not known to be methylated [197,198]. The CMV genome is not associated with histones when packaged in the virion [199,200], but becomes associated with histones within 30 min of entering a permissive fibroblast [201]. Some post-translational modifications to histones bound to DNA, such as acetylation, result in a more open chromatin structure for gene expression [202,203]. Methylation is more complex, with a single methylation of most residues serving as an activating mark, and accumulation of additional methyl groups tipping the balance toward repression on some histone residues but not others. Modifications to some residues are constitutively repressive whereas others are facultatively repressive and thus more readily reversible. The pattern of histone association during infection in permissive fibroblasts correlates with what is known about the dynamics of promoter activation and lytic gene expression. During immediate early times of infection, early and late promoters are associated with repressive histone marks; as the infection cycle progresses, early and then late promoters gradually become associated with histones carrying activating marks [204].

Because of its central role in initiation and progression of the viral replication cycle, much of the focus on chromatinization has centered on the MIEP. The MIEP is associated with relatively high levels of histone deacetylases (HDACs) and hypoacetylated histones during latent infection, consistent with a closed chromatin formation and less transcription from the locus [56,160,198]. Following differentiation into a permissive macrophage or dendritic cell, there is less association with HDACs and higher levels of histone acetylation [56,160,198]. In comparison to the transcriptionally repressed MIEP, the promoter that drives latency unique nuclear antigen (LUNA) expression is euchromatic during latent infection, indicating transcriptional activity [49]. Ioudinkova et al. demonstrated that the MIEP and representative early, early-late, and late promoters were preferentially associated with acetylated histone 3 at lysine 9 (H3K9) (a marker of activation for cellular genes) and not dimethylated H3K9 (a marker of repression) during lytic infection of differentiated THP-1 cells [81]. This correlated with detectable accumulation of IE mRNA and protein in these cells. However, during latent infection of THP-1 cells, the MIEP enhancer region was associated with neither activating acetylated H3K9 nor repressive dimethylated H3K9 histone modifications, although the early, early-late, and late promoters were associated with repressive dimethylation at H3K9 [81]. The finding that the MIEP enhancer is associated with neither activating nor repressive histone modifications while early and late promoters were repressed is consistent with reports that additional blocks downstream of IE expression exist as a barrier for viral reactivation [76]. This finding may also reflect a need for epigenetic modifications in the MIEP region to be readily reversible in response to changes in the cellular environment to allow viral reactivation to be triggered. This notion is consistent with studies in THP-1 and NT2 cells, which show that polycomb repressive complex 2 (PRC2), which is known to catalyze the formation of reversibly silenced facultative heterochromatin domains marked by histone H3 trimethylated at lysine 27 (H3K27me3), plays a role in regulating viral gene expression during latency [205].

It is presumed that the MIEP is activated due to a reversal in repressive chromatin following differentiation along the myeloid lineage into a permissive macrophage or dendritic cell. Treatment of quiescently infected NT2 cells with an HDAC inhibitor permits viral replication [192], and this same treatment results in disruption of repressive heterochromatin at the MIEP [198]. In latently infected NT2 cells, use of an HDAC inhibitor in combination with activation of the cAMP/PKA signaling pathway results in a synergistic increase in IE expression [188], indicating that a complex mechanism involving multiple cellular (and viral) factors is required to trigger viral reactivation. These changes correlate with increased levels of IE mRNA and protein, typically used as a proxy for MIEP activity. However, MIEP activity has not been explicitly demonstrated following reactivation. While it is clear that epigenetic regulation of the viral genome contributes to the establishment of, maintenance of, and reactivation from latency, further studies are needed to fully decipher the complex mechanisms that regulate the transition between latent and replicative states of infection.

### 5.3. Viral Factors and Chromatinization

During replicative infection, the MIEP is initially silenced following formation of a repressive chromatin structure by the nuclear domain 10/promyelocytic leukemia nuclear body (PML-NB) proteins Daxx and ATRX [200,204,206,207,208]. Early during infection in fibroblasts, the viral tegument protein pp71 traffics to the PML-NBs in the nucleus and displaces ATRX [209], then induces sumoylation [210] and ubiquitin-dependent degradation [207,211] of Daxx, resulting in IE expression [212,213,214,215,216,217]. After IE gene products are made, they further neutralize repression by PML-NB proteins and HDACs [200,206,207,218,219,220]. The PML-NB defense is also present in latency-associated NT2 and THP-1 cells, and could contribute to the establishment of latency [65,77]. In THP-1 cells, pp71 is sequestered in the cytoplasm, resulting in high levels of Daxx and repression of IEs [65]. IE and E gene expression is rescued following use of an HDAC inhibitor (VPA) or Daxx knockdown in NT2s and THP-1s infected with the AD169 lab strain of HCMV. However, the reactivation is ultimately aborted since viral L gene expression was not detected [65]. IE expression is not rescued by VPA treatment or Daxx knockdown during infection with clinical strains, suggesting a redundant mechanism in the ULb’ region for silencing IE expression during the establishment of latency [65]. One such factor is likely the viral latency determinant UL138, as its expression enhances the formation of repressive chromatin around the MIEP by inhibiting recruitment of lysine-specific demethylases (KDMs) [221] and represses viral replication for latency [91,92,222]. Incidentally, one of the KDMs whose function is blocked by UL138 has a specific binding activity to reverse facultative H3K27me3 histone modifications [223], suggesting a potential mechanism by which UL138 contributes to the maintenance of latency.

### 5.4. Viral Gene Expression during Latency

During latent infection in hematopoietic cells, it has been presumed that the viral genome is largely transcriptionally silent, particularly the MIEP, as early studies did not detect IE transcripts or proteins in latently infected cells [30,36,224]. This assertion is predominantly supported by transient transfection assays in undifferentiated NT2 and THP-1 cells [183,225] and by gene therapy studies where attempts to use the CMV MIE promoter have been unsuccessful [226]. More recent studies using highly sensitive techniques that can detect lower levels and a greater breadth of gene expression have shown that IE genes are transiently expressed at low levels following initial infection, and then silenced as latency is established [24,26,227,228]. While the gene expression detected could be attributed to infection in vitro and the heterogeneity of infected cells, these findings distinctly raise the possibility that low-level gene expression might exist in cells which are not productively replicating the virus [229]. Further blurring the lines of distinction between replicative and latent states of infection, binding of RNA polymerase II to the MIE region has been reported in latently infected cells [230] and transcripts originating from the MIE locus have been detected during both the establishment and maintenance of latency [27,64,229,231,232]. The true nature of this seemingly more dynamic latency is still somewhat obscured by the limitations inherent in using heterogeneous populations of hematopoietic cells that could potentially reflect different latency programs, such as those that are hallmarks of EBV latency [233].

These observations pose a series of new and important questions: if the MIEP is not completely silent during the latent infection, is transcription from the MIEP considerably reduced such that it sometimes falls below our limits of detection? Are there transient bursts of viral gene expression from the MIEP that only rarely reach a threshold required for the full reactivation of viral gene expression? Although IE transcripts are detected in the context of latent infection, there is no evidence that IE1 and IE2 proteins are synthesized or that this results in the production of progeny virions. Indeed, a recent study showed that ectopic expression of IE1 and IE2 in undifferentiated THP-1 cells is enough to activate early gene expression, but does not result in production of viral progeny [76], indicating that there are multiple blocks that must be navigated before full reactivation is triggered. An important one appears to be cellular differentiation, suggesting that a complex interaction of both viral and cellular factors is ultimately required for successful viral reactivation, as is seen in the alpha herpesvirus family [109,234].

The established model where reactivation of latent HCMV is triggered by differentiation along the myeloid lineage rests on the assumption that differentiation-dependent changes in the cellular environment trigger reactivation of the MIEP as a first step in initiating the viral gene expression program, although this has not been explicitly shown. A non-canonical IE1 transcript that includes exon 4, but not exons 1 and 2, was recently discovered in latently infected CD34+ cells [64]. This transcript arises from a cryptic promoter in the MIE locus, other than the MIEP, and encodes a smaller IE1 protein (IE1 exon 4) that is also expressed in latently infected CD34+ cells and is proposed to tether the latent viral genome to cellular chromatin to promote viral genome maintenance and amplification in dividing CD34+ cells [230]. This finding introduces the possibility that chromatin rearrangement during latency promotes silencing of the MIEP, but leaves alternative MIE promoters open and active to produce a latency-specific transcriptional profile. Further, other promoters have been identified to reside within intron A of the MIE gene locus that contribute to IE1 and IE2 expression in fibroblasts [235] and could contribute to reactivation in a cell-type-dependent manner. It remains to be determined if there is a true latent transcriptome or if gene expression is simply maintained at very low levels that do not support virus replication [236].

## 6. Final Thoughts

CMV latency is remarkably complex and has eluded definition due to the diversity of cell types infected in the host, the heterogeneity of cells harboring latent virus, the challenges of in vitro models, and the paucity of in vivo animal models. Going forward, it will be important for the field to embrace these complexities to understand how a multitude of virus–host interactions and the control of viral gene expression impact latency and reactivation. These include, but are not limited to, host pathways targeted by viral factors expressed during latency and the changes that accompany and, indeed, facilitate the transition from latent to active states of replication. As a field, we are well-poised with advancement of both in vitro and in vivo models and -omics technologies to make unprecedented leaps in our understanding of CMV latency and reactivation.
